# Optilume, a minimally invasive solution for BPH and urethral stricture: what we know, what we need? an EAU endourology scoping review

**DOI:** 10.1186/s12894-025-01896-3

**Published:** 2025-08-09

**Authors:** Vineet Gauhar, Steffi Kar Kei Yuen, Nariman Gadzhiev, Marcelo Wroclawski, Giacomo Maria Pirola, Ee Jean Lim, Angelo Cormio, Carlo Giulioni, Angelo Cafarelli, Dmitry Enikeev, Yunfu Liu, Jeremy Yuen Chun Teoh, Dean Elterman, Thomas Hermann, Daniele Castellani

**Affiliations:** 1 Department of Urology, Ng Teng Fong Hospital, Singapore, Singapore; 2 European Association of Urology Section of Endourology (ESEUT), Arnhem, The Netherlands; 3 Asian Institute of nephrourology, AINU, Hyderabad, India; 4 S. H. Ho Urology Centre, Department of Surgery, The Chinese University of Hong Kong, Hong Kong, China; 5 Department of Urology, Saint Petersburg State University Hospital, St. Petersburg, Russian Federation; 6 Hospital Israelita Albert Einstein, São Paulo, Brazil; 7 BP - a Beneficência Portuguesa de São Paulo, São Paulo, Brazil; 8 IRCCS Multimedica, Ospedale San Giuseppe, Milano, Italy; 9 Department of Urology, Singapore General Hospital, Academia Level 5, 20 College Rd, Singapore, 169856 Singapore; 10 Department of Urology, Azienda Ospedaliero-Universitaria delle Marche, Polytechnic University of Marche, Ancona, Italy; 11 Department of Urology, Casa di Cura Villa Igea, Ancona, Italy; 12 Rabin Medical Center (Belenson, Hasharon), Petah Tikva, Israel; 13 Faculty of Medicine, Tel Aviv University, Tel Aviv, Israel; 14 Department of Urology, Medical University of Vienna, Vienna, Austria; 15 Institute for Urology and Reproductive Health, Moscow, Russia; 16 The First Affiliated Hospital of School of Medicine, Zhejiang University, Hangzhou, China; 17 Li Ka Shing Institute of Health Sciences, The Chinese University of Hong Kong, Hong Kong, China; 18 Division of Urology, Department of Surgery, University of Toronto, Toronto, Canada; 19 Department of Urology, Kantonspital Frauenfeld, Spital Thurgau AG, Frauenfeld, Switzerland; 20 Urology Unit, Azienda Ospedaliero Universitaria Delle Marche, Ancona, Italy

**Keywords:** Optilume, Optilume BPH, Benign prostatic hyperplasia, Urethral stricture, Lower urinary tract symptoms

## Abstract

**Introduction:**

Optilume and Optilume BPH, a minimally invasive drug-coated balloon (DCB) combining mechanical dilation with paclitaxel delivery, offers a novel approach for treating urethral strictures and benign prostatic hyperplasia (BPH) respectively. This scoping review summarizes current evidence on their efficacy, safety, and long-term outcomes to evaluate their role in reducing recurrence and improving patient-reported and functional outcomes.

**Methods:**

Following PRISMA guidelines, a systematic search (Embase, PubMed, Cochrane, Scopus) until March 2025 identified 287 studies. Eligibility followed PICOS criteria, excluding non-English articles, reviews, and case reports. Risk of bias was assessed using Cochrane RoB 2 and MINORS tools. Data extraction focused on anatomical success, symptom improvement, complications, and retreatment rates. This review was registered at https://osf.io/vf4dw.

**Results:**

After screening, 20 studies met inclusion criteria: 2 preclinical animal studies, 12 clinical studies on urethral strictures, and 6 on BPH. For urethral strictures, the ROBUST trials demonstrated 71.7% freedom from reintervention at 5 years, with sustained improvements in peak flow rate (Qmax: 5.0 to 19.9 mL/s) and IPSS (25.2 to 7.2). In BPH, the PINNACLE trial reported a 67.5% responder rate (≥ 30% IPSS improvement) at 2 years, with IPSS reduced from 23.4 to 11.0. Qmax improved from 8.9 to 19.0 mL/s, and sexual function (IIEF scores) remained stable. Safety profiles were favorable, with transient hematuria (15–39.8%) and no severe complications. Cost analyses indicated potential savings due to reduced retreatment.

**Conclusion:**

Optilume provides significant symptom relief for BPH and urethral strictures, with low recurrence rates and preserved sexual function. Its minimally invasive nature, combined with targeted drug delivery, positions it as a promising alternative to traditional surgeries. Further research is needed to expand indications and validate long-term outcomes and cost-effectiveness across diverse populations.

**Supplementary Information:**

The online version contains supplementary material available at 10.1186/s12894-025-01896-3.

## Introduction

Optilume is an innovative, minimally invasive treatment that utilizes a drug-coated balloon (DCB) technology to address two common urological conditions: Benign prostatic hyperplasia (BPH) and urethral strictures. It offers a unique approach by combining mechanical dilation with targeted drug delivery to provide immediate relief. In BPH its role as a minimally invasive surgical therapy (MIST) and for urethral stricture its utility as a better alternative to urethral dilation and direct vision internal urethrotomy (DVIU) are being evaluated [1–3].

**Fig. 1:**
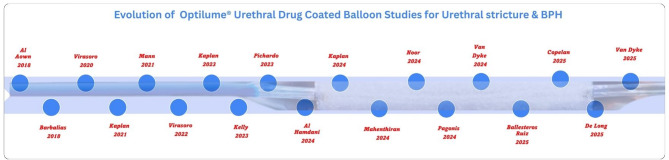
Timeline showing the evolution of Optiume Urethral Drug Coated Balloon. BPH- benign prostatic hyperplasia

Our scoping review explores the entire landscape from experimental to current clinical evidence for its use in urethral stricture and BPH and delve into insights that promise how the indications for this intervention can be expanded and potential utility in other urological conditions (Fig. 1).

## Evidence acquisition

### Literature search

This review followed the PRISMA extension for scoping reviews [4]. Literature search was performed on 31 st March 2025 from inception using Embase, PubMed, Cochrane Central Register of Controlled Trials, and Scopus. The following term and Boolean operators were used (“benign prostatic hyperplasia” OR BPH OR “urethral stricture” OR “ureteral stenosis”) AND (Optilume OR “paclitaxel-coated balloon” OR “drug-coated balloon” OR DCB).

### Selection criteria

The PICOS (Patient, Intervention, Comparison, Outcome, Study type) framework was used to frame and answer the clinical question: P: adults with LUTS due to BPH or urethral stricture or animal studies; I: treatment with Optilume; C: none or any other treatment; O: complications and functional outcomes; S: retrospective, prospective, and randomized.

### Study screening and selection

Studies were accepted based on PICOS eligibility criteria. Non English papers were excluded. Reviews, letters to the editor, meeting abstracts, and case reports were also excluded. Retrospective studies, prospective studies, and prospective randomized studies were accepted. The quality assessment of the included studies was performed using the Cochrane Risk of Bias tools, using RoB 2 for randomized studies [5]. Risk of bias in single-arm studies was evaluated using the methodological index for non-randomized studies (MINORS) instrument, a 12-item instrument built to evaluate the methodological quality of non-randomized surgical studies, with each score ranging from 0 to 2 (the higher the score, the better the quality of the study) [6].

Two independent authors screened all the retrieved studies using Covidence systematic review software (Veritas Health Innovation, Melbourne, Australia). Discrepancies were solved by consultation. The full text of the screened papers was selected if found pertinent to the aim of this review. This review was registered at https://osf.io/vf4dw.

## Results

**Fig. 2:**
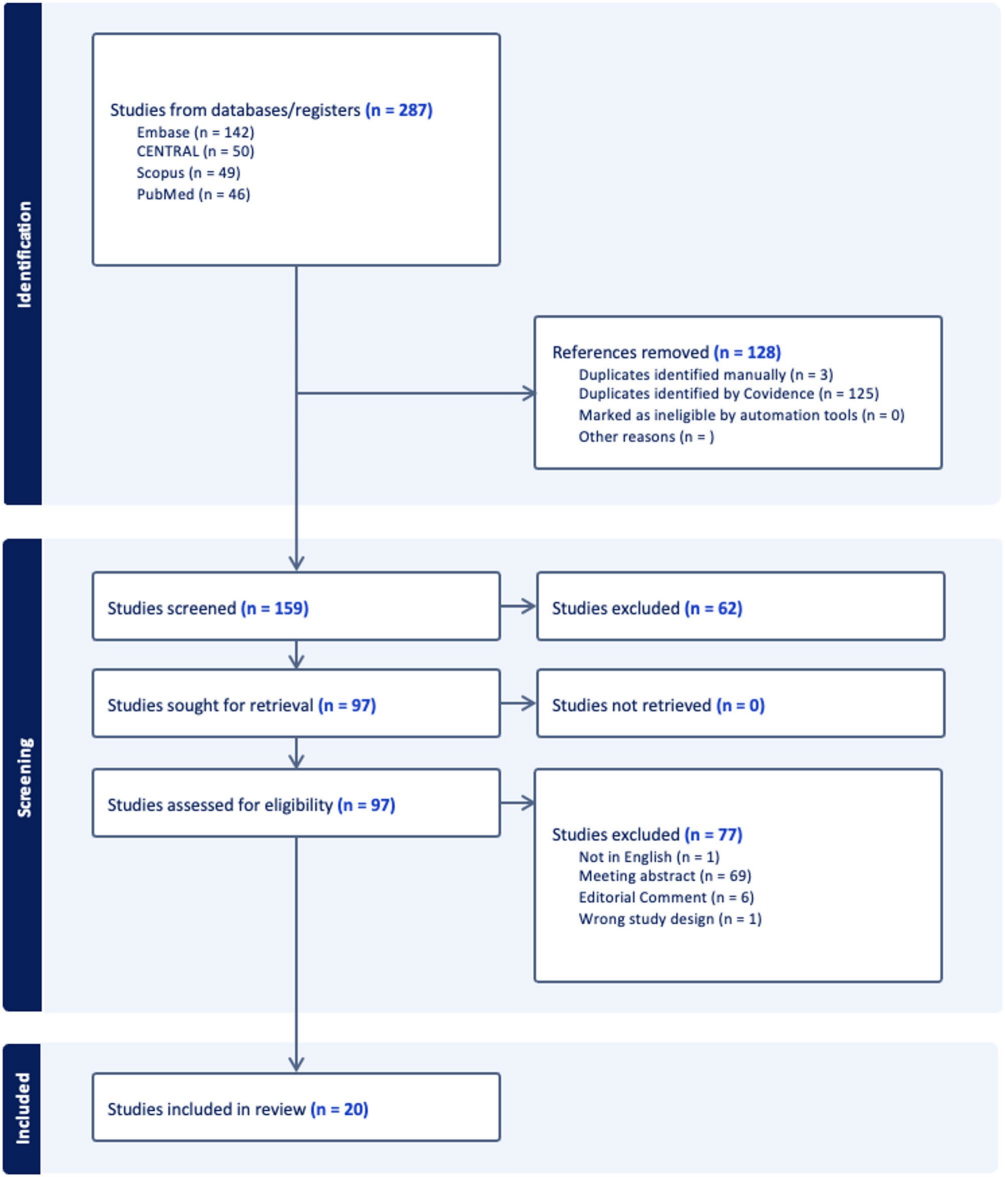
PRISMA flow diagram of the literature search

Literature search gathered 287 papers. 128 duplicates were excluded. 159 papers were left and screened against title and abstract. 62 papers were further excluded because they did not meet the inclusion criteria. The remaining 97 full-text papers were screened for relevance and 77 papers were excluded. Finally, 20 papers were accepted and included with 2 papers dealing with animal studies [1, 7], 12 papers reporting treatment in urethral stricture [2, 3, 8–17] and the remaining ones dealing with treatment of clinical BPH [18–23]. Figure 2 shows the PRISMA flow diagram of the literature search.

### Study characteristics

Table 1 shows the characteristics of included studies. An animal study assessed the distribution of Paclitaxel in the rabbit urethra [1] and a further the effect of Paclitaxel on the wall of strictured rabbit’s urethra [7]. There were two trials assessing the safety and efficacy of optilume in clinical BPH, namely the EVEREST-I study (a prospective, single-arm, non-randomized, open label, multicenter study) [20, 21, 23], and the PINNACLE study (a double-blind, multicenter, randomized, sham-controlled study) [18, 19, 22]. Regarding urethral stricture, there were a single-center, multicenter, prospective, open-label trial (ROBUST I study) [8, 10–12], a multicenter, prospective, randomized trial (ROBUST III study) [14, 15], two prospective, single center studies [2, 13], two retrospective multicenter studies [9, 16] and a retrospective single-center study [17]. A further study sought to assess the economic evaluation of the optilume compared with endoscopic management of recurrent anterior urethral stricture in England [3]

**Table 1:** Characteristics of included studies

Author	Published Year	Type of Study	Aim of study
Optilume – experimental studies			
Barbalias [1]	2018	Animal study	Paclitaxel was distributed to the urothelial, submucosal, and smooth muscle layers of the normal rabbit urethra immediately after dilatation with a DCB. Paclitaxel and mild inflammation were present at the site 24 and 48 h after the dilatation.
Pagonis [7]	2024	Animal	Paclitaxel’s enrichment was detected in the smooth muscle layer of all specimens that have been harvested immediately and 24 h after the dilation with Advance 18 Paclitaxel PTA balloons. Paclitaxel may play an inhibitive role in the recurrence of the stenosis.
Optilume – clinical studies			
Virasoro [12]	2020	prospective, single arm	One-year data indicates the Optilume paclitaxel-coated balloon is safe for the treatment of recurrent bulbar urethral strictures. Early efficacy results are encouraging and support further followup of these men through five years, as well as further investigation with a randomized trial.
Elliot [14]	2021	RCT	The results of this randomized controlled trial support that Optilume is safe and superior to standard direct vision internal urethrotomy/dilation for the treatment of recurrent anterior urethral strictures < 3 cm in length. The Optilume DCB may serve as an important alternative for men who have had an unsuccessful direct vision internal urethrotomy/dilation but want to avoid or delay urethroplasty.
Mann [11]	2021	prospective, single arm	Two-year data indicates the Optilume PCB is safe for the treatment of recurrent bulbar urethral strictures. Early efficacy results are encouraging and support further followup of these men through five years, as well as further investigation with a randomized trial.
Virasoro [8]	2022	prospective, single arm	Symptomatic improvement after treatment with the Optilume DCB was maintained through 3 years in a population highly susceptible to recurrent urethral stricture disease. This minimally invasive therapy is safe with no negative impact on sexual function.
Kelly [3]	2023	Markov model for cost analysis	The analysis suggests that the Optilume urethral DCB treatment can be a cost-saving alternative management option for the treatment of recurrent anterior male urethral stricture within the NHS in England.
Mahenthiran [17]	2024	Retrospective cohort	DCB offers a safe and less invasive treatment for both treatment-naïve and recurrent urethral strictures with paclitaxel coating to prevent recurrence. Repeat intervention was not required for 90.7% of our cohort within an average follow-up duration of 9 months postoperatively. As DCB grows in clinical use, investigation into its long-term efficacy is justified.
Noor [13]	2024	prospective, single arm	It is concluded that Optilume balloon dilatation represents a promising therapeutic approach for urethral stricture, as evidenced by significant improvements in IPSS, Qmax, and PVR within 30 days post-procedure.
VanDyke [15]	2024	RCT	The Optilume DCB shows sustained improvement in both objective and subjective voiding parameters at 2-year follow-up. Optilume appears to provide a safe and effective endoscopic treatment alternative for short recurrent anterior urethral strictures among men who wish to avoid or delay formal urethroplasty.
Alhamdani [2]	2024	prospective, single arm	76% were stricture free at 30 months follow-up with no complications.
VanDyke [16]	2025	Retrospective comparative	Despite a greater number of prior surgical interventions, patients with a history of urethroplasty achieve similarly high success rates after treatment with DCB compared to those without a history of urethroplasty.
Ballesteros Ruiz [9]	2025	Retrospective cohort	The treatment success rate was 73.8%, with a median follow-up of 8 months.
DeLong [10]	2025	prospective, single arm	Optilume DCB shows sustained improvement in subjective and objective voiding parameters at 5 years. Optilume is a safe and effective treatment option for appropriately selected men with recurrent bulbar urethral stricture who wish to avoid urethroplasty.
Optilume BPH – clinical studies			
Kaplan [20]	2021	Prospective single arm	Treatment with the minimally invasive Optilume BPH Catheter System is safe and showed subjective and objective improvements in LUTS. Benefits were rapid and persisted through 1 year. The initial results warrant further evaluation of this therapy as a treatment option for patients with LUTS related to BPH.
Pichardo [21]	2023	Prospective single arm	The Optilume BPH Catheter System combines mechanical and pharmaceutical aspects for the treatment of BPH, resulting in both an immediate functional improvement by way of creating an anterior commissurotomy and a sustained anatomic result due to the application of paclitaxel to the prostatic urethra during the dilation. The functional and symptomatic improvements seen after treatment with Optilume BPH are significant and have been sustained through 2 years in this early feasibility study.
Kaplan [19]	2023	RCT	Treatment with Optilume BPH provides immediate and sustained improvements in obstructive symptoms and flow rate while preserving erectile and ejaculatory function. Treatment is well tolerated and can be done in an office or ambulatory setting.
Kaplan [22]	2024	RCT	In the PINNACLE study, participants treated with the Optilume BPH Catheter System demonstrated continued and durable results at 2 years, affirming tolerability, safety, and the enduring effectiveness. The Optilume BPH Catheter System provides lasting results that are comparable to the more invasive therapies, while preserving the advantages with being a minimally invasive therapy.
Kaplan [23]	2024	Prospective single arm	Long-term followup through four years for subjects treated with the Optilume BPH Catheter System indicates durable outcomes in symptom improvement and functional improvement in flow rate. These results indicate the unique mechanism of action for Optilume BPH successfully achieves an immediate mechanical effect that is maintained long-term through incorporation of paclitaxel to maintain patency.
Copelan [18]	2025	RCT	Optilume BPH appears to provide clinical benefit with a high degree of patient satisfaction and minimal impact on sexual function.

*DCB* drug-coated balloon, *BPH* benign prostatic hyperplasia

### Risk of bias assessment

Supplementary Fig. 1 shows the details of quality assessment for the randomized clinical trials that showed some concerns regarding overall risk bias due to missing of outcome data. Supplementary Table 1 shows the details of quality assessment of the single-arm studies.

The highest MINORS score was 18, whilst the lowest score was 9.

## Discussion

### Mechanism of action of Optilume: experimental studies

Male urethral stricture is a common condition caused by injury to the urethra, leading to obstructive symptoms and impacting quality of life. Progressive dilatation is simple but often results in high re-stenosis rates due to type 3 collagen production [24]. Paclitaxel (PTX), a cytostatic agent known for its antiproliferative properties, may help prevent recurrence by reducing RNA expression of key fibrogenic genes including TGF-β1, CTGF, and PAI-1 [25]. However, effective PTX delivery requires disrupting the urothelial lining for deeper penetration Hence, DCBs have been developed for this purpose [26].

Barbalias et al. studied PTX distribution in rabbit urethras after DCB dilation [1]. Standard balloon dilation only caused urothelial denudation, while DCBs allowed PTX penetration through all layers. Histological exams showed mild inflammation initially and limited drug diffusion due to PTX’s lipophilic nature. Yet, at six weeks, DCB dilation decreased inflammation more rapidly, confining fibrosis to the epithelial layer, suggesting PTX’s potential to inhibit fibrotic processes [7], therein preventing a fibrotic recurrence.

### Optilume: clinical studies

The Optilume urethral DCB is inserted into the urethra and inflated at the site of the stricture to mechanically dilate the narrowed passage. Simultaneously, the balloon delivers PTX directly to the surrounding tissue. The antiproliferative properties of paclitaxel help to inhibit the formation of new scar tissue, thereby reducing the risk of stricture recurrence.

The ROBUST trials have systematically outlined the immediate and 5 year outcomes of this intervention. ROBUST I, II, and III clinical trials provide comprehensive evidence supporting the efficacy, safety, and durability of the Optilume DCB for the treatment of anterior urethral strictures [14, 15, 27]. In Robust I, 12 months, anatomic success was achieved in 70% of the 53 males with < 12Fr, <2 cm anterior urethral stricture with 2-year sustained improvements in Qmax and international index of erectile function (IIEF) satisfaction scores. At 5 years, 71.7% of patients did not need re-treatment with long-term improvements in International Prostate Symptom Score (IPSS) and Qmax maintained. No new safety concerns were reported during extended follow-up (Table 2).

**Table 2:** ROBUST I: key outcomes over 5 years

Outcome	Baseline Virasoro et al., 2020 [12]	1-Year Virasoro et al., 2020 [12]	2-Year Mann et al., 2021 [11]	3-Year Virasoro et al., 2022 [8]	5-Year DeLong et al., 2025 [10]
Freedom from reintervention	—	83% (40/48)	82% (38/46)	77% (33/43)	71.7%
Anatomic Success (%)	—	70% (37/53)	70% (32/46)	67% (29/43)	58% (25/43)
IPSS	25.2 ± 4.46	4.9 ± 5.63	6.9 ± 7.66	5.5 ± 6.9	7.2 ± 6.6
IPSS QoL	4.9 ± 0.86	0.8 ± 1.06	0.9 ± 1.47	0.7 ± 1.2	0.7 ± 1.2
Qmax (mL/s)	5.0 ± 2.56	19.5 ± 9.96	17.5 ± 10.39	15.1 ± 8.3	19.9
PVR (mL)	141.4 ± 105.05	26.79 ± 33.1	45.5 ± 49.5	50.2 ± 62.5	59.5
IIEF	6.5 ± 2.62	7.8 ± 2.62	7.6 ± 2.48	8.2 ± 2.2	8.3 ± 1.8
Complication rate (%)	—	5.7% (Clavien I–II only)	No new complications	No new complications	No new complications

*IPSS* international prostate symptom score, *QoL* quality of life, *PVR* post void residual urine, *IIEF* international index of erectile function

### Clinical studies of Optilume BPH

The Optilume BPH Catheter System represents a groundbreaking minimally invasive treatment for lower urinary tract symptoms (LUTS) due to BPH, integrating mechanical dilation with paclitaxel, an antiproliferative agent known for preventing restenosis in vascular applications, inhibits scar tissue formation and prostatic tissue growth to enhance symptom relief and durability [20].

Unlike traditional balloon dilation methods that lacked sustained efficacy due to tissue recovery [28], Optilume provides immediate and long-lasting benefits [20, 21]. Clinical evidence from the EVEREST-I and PINNACLE studies underscores its efficacy, demonstrating significant improvements in LUTS across diverse patient cohorts [21, 22]. In the EVEREST-I study, a single-arm trial with 80 men, 81.3% of subjects achieved a ≥ 40% reduction in IPSS at 3 months, with IPSS dropping from 22.3 to 7.9 at 1 year and stabilizing at 8.2 at 2 years [20, 21].

The PINNACLE study, a randomized controlled trial involving 148 men, reported a 67.5% symptomatic responder rate (≥ 30% IPSS improvement) at 2 years, with IPSS improving from 23.4 to 11.0. These positive results from symptomatic responders, though not a standardized metric of BPH outcome, highlighted rapid symptom relief within weeks, sustained through 2 years.

Objective improvements in urinary function further validate Optilume’s effectiveness. In EVEREST-I, peak urinary flow rate (Qmax) rose from 10.9 to 18.4 mL/s at 1 year and 17.2 mL/s at 2 years [21], while PINNACLE showed an increase from 8.9 to 19.0 mL/s at 2 years, exceeding typical MIST improvements of 4–5 mL/s [21, 22]. Post-void residual (PVR) volume also decreased, from 63.1 to 33.9 mL at 1 year in EVEREST-I and from 83.7 to 65.9 mL at 2 years in PINNACLE, indicating enhanced bladder emptying [22].

Quality of life (QoL) measures improved significantly, with EVEREST-I showing IPSS QoL dropping from 4.6 to 1.3 at 1 year and 1.6 at 2 years, and BPH Impact Index (BPH-II) from 6.9 to 2.0 and 2.3, respectively [20, 21]. In PINNACLE, BPH-II improved from 7.0 to 2.3 at 2 years, a 53.9% enhancement, reflecting substantial QoL gains [22]. These subjective and objective outcomes position Optilume as a robust alternative to conventional therapies.

Safety profiles from both studies indicate Optilume is well-tolerated. EVEREST-I reported common urologic adverse events (AEs) like post-procedural hematuria (15.0%) and urinary tract infections (8.8%), mostly mild and resolving within 3 months, with no treatment-related AEs after 12 months. PINNACLE noted hematuria (39.8%) and urinary tract infections (11.2%), with no serious device-related AEs beyond 12 months, aided by a hematuria management protocol [20–22]. Two issues however need to be highlighted; first, lower improvement in storage symptoms among non-responders suggest that potential irritative factors such as detrusor overactivity need to be carefully addressed, thus highlighting the role of urodynamics in patient selection. Second, the Everest studies included only patients with prostate volumes under 80 mL and there was no control group; therefore, concerns about methodology as well as applicability of the findings to broader patient populations should be acknowledged.

Adjustments in device sizing, such as removing larger balloons in EVEREST-I, reduced complications like bleeding (from 54.5 to 19.0%), enhancing safety profile. Pharmacokinetic data showed minimal systemic paclitaxel exposure, with low plasma and semen concentrations, supporting its localized action and safety [20].

A key advantage of Optilume is its preservation of sexual function, a critical concern with surgical options like transurethral resection of prostate (TURP) [18, 22]. In PINNACLE, IIEF and Male Sexual Health Questionnaire-Ejaculatory Dysfunction (MSHQ-EjD) scores remained stable or slightly improved at 12 months compared to sham, with IIEF rising from 15.6 to 20.4 at 2 years [22]. EVEREST-I confirmed no significant changes in sexual function through 2 years, contrasting with TURP’s risks of retrograde ejaculation. This preservation enhances patient satisfaction and QoL, addressing a major drawback of invasive treatments [21]. In fact, Copelan et al. [18] showed that in the 148 patients they treated, the IIEF and MSHQ-EjD scores remained stable at 12 months, even in patients with pre-existing dysfunction. Moreover, semen parameters showed no significant alterations.

Overall, excellent IPSS reduction and sexual function shows that such findings are not only comparable but perhaps best in its category of non resection-based MISTs, making the Optilume BPH Catheter System strongly appropriate for sexually active patients aiming to keep their sexual functions [29, 30]. Limitations include restricted prostate size (< 80 g) and exclusion of significant median lobes, necessitating further research for broader applicability [21, 22].

### Real world expanded indications and potential applications

The multi-institutional Optilume registry (NCT05479422), funded by the European Association of Urology (EAU) Research Foundation, is vital for collecting real-world data [31]. Optilume has shown promise as a treatment for urethral strictures, but its application in ureteral strictures remains investigational. The primary potential benefit of Optilume in treating ureteral strictures lies in its ability to reduce recurrence rates compared to standard endoscopic dilation, due to its antiproliferative properties. Future studies are required to determine its safety and efficacy across various etiologies, such as post-surgical, inflammatory, and idiopathic cases.

A recent study assessed the outcomes of upfront Optilume DCB dilation in patients with complex recurrent urethral stricture disease who had failed at least two prior dilatations and presented with urinary retention. Results indicated that 65.4% (17/26) of subjects voided satisfactorily and were free of recurrence and re-operation [32]. Additionally, the first Optilume urethral DCB was used for female urethral stricture involving the sphincter, showing that the patient remained stricture-free with full continence and complete bladder emptying at six months follow-up [33].

The ROBUST trial proposed treatment for anterior urethral strictures, and a retrospective single-centre study involving 43 patients with any type of urethral stricture reported a nine-month intervention-free follow-up in 90.7% of cases [17]. Furthermore, a real-world Spanish multicentre study including 238 patients showed a 73.8% success rate in 156 cases with a median follow-up of eight months. Authors concluded that recurrence rates were higher in strictures located in the posterior versus anterior urethra (42.9% vs. 24.6%, *p* = 0.126). No significant differences were observed between patients with and without prior urethral manipulation [9].

To assess the economic impact of BPH treatments, it is vital to consider both initial procedure costs and long-term follow-up. Treatments with lower initial costs may have higher follow-up expenses due to retreatment or complications. Optilume’s economic cost for BPH and urethral stricture includes device cost, procedure expenses, potential savings from fewer reinterventions, and comparisons to standard treatments. This can vary by healthcare system, hospital, and patient factors. Further analysis is needed, but Optilume could offer cost benefits over more invasive surgeries considering long-term outcomes and quality of life [3].

The current review holds several limitations warranting acknowledgement, including potential selection bias in the included studies, as well as small sample sizes and a short follow-up period of the included studies.

## Conclusion

Literature demonstrates that this drug-coated balloon technology provides a valuable minimally invasive alternative, improving patient outcomes and reducing the need for repeat interventions. Optilume’s favourable safety profile and utility for both BPH and urethral strictures from studies like PINNACLE and ROBUST trials highlight significant symptom improvement with minimal adverse events upon 2 years, and 5 years follow-up respectively. For BPH, it offers a less invasive option preserving sexual function. In urethral strictures it reduces recurrence rates leading to potential long-term cost-effectiveness.

## Supplementary Information

Supplementary Material 1.

Supplementary Material 2.

Supplementary Material 3.

## Data Availability

Data will be shared by the corresponding author upon reasonable request.

## References

[1] Barbalias D, Lappas G, Ravazoula P, et al. Evaluation of the distribution of Paclitaxel after application of a Paclitaxel-Coated balloon in the rabbit urethra. J Endourol. 2018;32(5):381–6. 10.1089/end.2017.0935. 29382215 10.1089/end.2017.0935

[2] Alhamdani Z, Ong S, Zhong W, Chin P. Optilume((R)) Drug-Coated balloon May lower the Re-Treatment rate postintervention for challenging urethral stricture disease in Long-Term Follow-Up: A prospective cohort study. J Endourol. 2024;38(11):1192–200. 10.1089/end.2024.0318. 39119807 10.1089/end.2024.0318

[3] Kelly L, Shore J, Wright J, Patrick C, Holmes H. Economic evaluation of optilume, a drug-coated balloon for recurrent anterior male urethral stricture. BJUI Compass. 2023;4(4):430–6. 10.1002/bco2.241. 37334026 10.1002/bco2.241 PMC10268567

[4] Tricco AC, Lillie E, Zarin W, et al. PRISMA extension for scoping reviews (PRISMA-ScR): checklist and explanation. Ann Intern Med. 2018;169(7):467–73. 10.7326/M18-0850. 30178033 10.7326/M18-0850

[5] Sterne JAC, Savovic J, Page MJ, et al. RoB 2: a revised tool for assessing risk of bias in randomised trials. BMJ. 2019;366:l4898. 10.1136/bmj.l4898. 31462531 10.1136/bmj.l4898

[6] Slim K, Nini E, Forestier D, et al. Methodological index for non-randomized studies (minors): development and validation of a new instrument. ANZ J Surg. 2003;73(9):712–6. 10.1046/j.1445-2197.2003.02748.x. 12956787 10.1046/j.1445-2197.2003.02748.x

[7] Pagonis K, Peteinaris A, Adamou C, et al. Minimal invasive treatment of urethral strictures: an experimental study of the effect of Paclitaxel coated balloons in the wall of strictured rabbit’s urethra. Arch Ital Urol Androl. 2024;96(1):12248. 10.4081/aiua.2024.12248. 38389459 10.4081/aiua.2024.12248

[8] Virasoro R, DeLong JM, Estrella RE, et al. A Drug-Coated balloon treatment for urethral stricture disease: Three-Year results from the ROBUST I study. Res Rep Urol. 2022;14:177–83. 10.2147/RRU.S359872. 35572815 10.2147/RRU.S359872 PMC9091705

[9] Ballesteros Ruiz C, Campos-Juanatey F, Povo Martin I, et al. Efficacy and safety of Optilume(R) paclitaxel-coated urethral dilatation balloon in real-life: experience in a Spanish multicenter study. Actas Urol Esp (Engl Ed). 2025;49(1):80–5. 10.1016/j.acuroe.2024.10.003. 39486795 10.1016/j.acuroe.2024.10.003

[10] DeLong J, Virasoro R, Pichardo M, et al. Long-Term outcomes of recurrent bulbar urethral stricture treatment with the optilume Drug-Coated balloon: Five-Year results from the ROBUST I study. J Urol. 2025;213(1):90–8. 10.1097/JU.0000000000004229. 39213367 10.1097/JU.0000000000004229 PMC12708045

[11] Mann RA, Virasoro R, DeLong JM, et al. A drug-coated balloon treatment for urethral stricture disease: Two-year results from the ROBUST I study. Can Urol Assoc J. 2021;15(2):20–5. 10.5489/cuaj.6661. 32744999 10.5489/cuaj.6661 PMC7864702

[12] Virasoro R, DeLong JM, Mann RA, et al. A drug-coated balloon treatment for urethral stricture disease: interim results from the ROBUST I study. Can Urol Assoc J. 2020;14(6):187–91. 10.5489/cuaj.6323. 31977303 10.5489/cuaj.6323 PMC7654665

[13] Noor S, Abdullah A, Uroos SU, et al. Outcomes of optilume balloon dilatation in patient with urethral stricture. Pakistan J Med Health Sci. 2024;18(8):2. 10.53350/pjmhs020241881.

[14] Elliott SP, Coutinho K, Robertson KJ, et al. One-Year results for the ROBUST III randomized controlled trial evaluating the Optilume((R)) Drug-Coated balloon for anterior urethral strictures. J Urol. 2022;207(4):866–75. 10.1097/JU.0000000000002346. 34854748 10.1097/JU.0000000000002346 PMC12721643

[15] VanDyke ME, Morey AF, Coutinho K, et al. Optilume drug-coated balloon for anterior urethral stricture: 2-year results of the ROBUST III trial. BJUI Compass. 2024;5(3):366–73. 10.1002/bco2.312. 38481667 10.1002/bco2.312 PMC10927926

[16] VanDyke M, Joshi E, Ceballos B, et al. Efficacy of the optilume Paclitaxel drug-coated balloon after urethroplasty: short-term results from a multicenter study. Ther Adv Urol. 2025;17:17562872241312522. 10.1177/17562872241312522. 39840118 10.1177/17562872241312522 PMC11748073

[17] Mahenthiran AK, Burns RT, Soyster ME, et al. A single-institution experience with the optilume urethral drug coated balloon for management of urethral stricture disease. Transl Androl Urol. 2024;13(8):1498–505. 10.21037/tau-24-104. 39280647 10.21037/tau-24-104 PMC11399038

[18] Copelan O, Moss J, Freedman S, et al. Preservation of sexual function with Optilume-a novel treatment for lower urinary tract symptoms secondary to benign prostatic hyperplasia. J Sex Med. 2025;22(3):446–53. 10.1093/jsxmed/qdae206. 39829245 10.1093/jsxmed/qdae206

[19] Kaplan SA, Moss J, Freedman S, et al. The PINNACLE study: A Double-blind, randomized, Sham-controlled study evaluating the optilume BPH catheter system for the treatment of lower urinary tract symptoms secondary to benign prostatic hyperplasia. J Urol. 2023;210(3):500–9. 10.1097/JU.0000000000003568. 37555604 10.1097/JU.0000000000003568 PMC12721621

[20] Kaplan SA, Pichardo M, Rijo E, et al. One-year outcomes after treatment with a drug-coated balloon catheter system for lower urinary tract symptoms related to benign prostatic hyperplasia. Prostate Cancer Prostatic Dis. 2021;24(4):1073–9. 10.1038/s41391-021-00362-z. 33833379 10.1038/s41391-021-00362-z PMC8616755

[21] Pichardo M, Rijo E, Espino G, et al. Durable benefit after treatment of obstructive benign prostatic hyperplasia with a novel drug-device combination product: 2-year outcomes from the EVEREST-I study. World J Urol. 2023;41(8):2209–15. 10.1007/s00345-023-04473-1. 37354260 10.1007/s00345-023-04473-1

[22] Kaplan SA, Moss JL, Freedman SJ. Two-year long-term follow-up of treatment with the optilume BPH catheter system in a randomized controlled trial for benign prostatic hyperplasia (The PINNACLE Study). Prostate Cancer Prostatic Dis. 2024;27(3):531–6. 10.1038/s41391-024-00833-z. 38684918 10.1038/s41391-024-00833-z PMC11319191

[23] Kaplan SA, Pichardo M, Rijo E, et al. Long-term outcomes after treatment with optilume BPH Four-year results from the EVEREST study. Can Urol Assoc J. 2024;18(11):E319–25. 10.5489/cuaj.8737. 38976898 10.5489/cuaj.8737 PMC11534392

[24] Yang Y, Yu B, Sun D, Wu Y, Xiao Y. The dose-dependence biological effect of laser fluence on rabbit fibroblasts derived from urethral Scar. Lasers Med Sci. 2015;30(3):1019–29. 10.1007/s10103-014-1683-4. 25388915 10.1007/s10103-014-1683-4

[25] Kurniawan W, Soesatyo M, Aryandono T. The effects of docetaxel and/or Captopril in expression of TGF-beta1, MMP-1, CTGF, and PAI-1 as markers of anterior urethral stricture in an animal model. Ther Adv Urol. 2020;12:1756287220927994. 10.1177/1756287220927994. 35173811 10.1177/1756287220927994 PMC8842176

[26] Bayne DB, Gaither TW, Awad MA, et al. Guidelines of guidelines: a review of urethral stricture evaluation, management, and follow-up. Transl Androl Urol. 2017;6(2):288–94. 10.21037/tau.2017.03.55. 28540238 10.21037/tau.2017.03.55 PMC5422698

[27] DeLong JM, Ehlert MJ, Erickson BA, Robertson KJ, Virasoro R, Elliott SP. One-Year outcomes of the ROBUST II study evaluating the use of a Drug-Coated balloon for treatment of urethral stricture. Société Int d’Urologie J. 2022;3(1):21–7. 10.48083/MLXK5817.

[28] Gravas S, Malde S, Cornu JN, et al. From BPH to male LUTS: a 20-year journey of the EAU guidelines. Prostate Cancer Prostatic Dis. 2024;27(1):48–53. 10.1038/s41391-023-00700-3. 37488274 10.1038/s41391-023-00700-3

[29] Roehrborn CG, Rukstalis DB, Barkin J, et al. Three year results of the prostatic urethral L.I.F.T. Study. Can J Urol. 2015;22(3):7772–82. 26068624

[30] McVary KT, Roehrborn CG. Three-Year outcomes of the prospective, randomized controlled Rezum system study: convective radiofrequency thermal therapy for treatment of lower urinary tract symptoms due to benign prostatic hyperplasia. Urology. 2018;111:1–9. 10.1016/j.urology.2017.10.023. 29122620 10.1016/j.urology.2017.10.023

[31] Oszczudlowski M, Bialek L, Vetterlein MW, Trauma, Reconstructive Urology Working Party of the European Association of Urology Young Academic U. Paclitaxel-coated balloon dilation for urethral stricture disease: 5 years of clinical insights and future directions for optilume. Eur Urol Focus. 2025. 10.1016/j.euf.2025.03.013. 40175242 10.1016/j.euf.2025.03.013

[32] Jelisejevas LA, Rehder P, Wassermann J, Kink P, Tulchiner G. Optilume Drug-Coated balloon for acute urinary retention after failed treatment for complex recurrent urethral stricture disease. Medicina. 2025;61(4):700. 10.3390/medicina61040700. 40282993 10.3390/medicina61040700 PMC12028934

[33] Stuehmeier J, Jelisejevas LA, Kink P, et al. Optilume(R) drug-coated balloon dilation in complex female urethral stricture. Urol Case Rep. 2022;41:101987. 10.1016/j.eucr.2021.101987. 35070722 10.1016/j.eucr.2021.101987 PMC8766557

