# Supplementary material for: Optilume, a minimally invasive solution for BPH and urethral stricture: what we know, what we need? an EAU endourology scoping review
Source: BMC Urol. 2025 Aug 9;25:196. doi: 10.1186/s12894-025-01896-3 (PMC12335074; doi:10.1186/s12894-025-01896-3)
Supplement: Supplementary file 1 — Supplementary Material 1. [file 12894_2025_1896_MOESM1_ESM.docx]

**Supplementary Table 1**. Quality of included studies assessed using the MINORS (Methodological Index for NOn-Randomized Studies) score.

| **Author, year** | A stated aim of the study | Inclusion of consecutive  patients | Prospective collection of data | Endpoints appropriate to the aim of the study | Unbiased assessment of the study endpoint | Follow-up period appropriate  to the major endpoint | Loss to follow up not  exceeding 5% | Prospective calculation of study size | An adequate control group | Contemporary groups   \|  \| \| --- \| | Baseline equivalence of groups | Adequate statistical analyses | **Total score** |
| --- | --- | --- | --- | --- | --- | --- | --- | --- | --- | --- | --- | --- | --- | --- |
| EVEREST-I | 2 | 2 | 2 | 2 | 1 | 2 | 1 | 2 | 0 | 2 | 0 | 2 | **18** |
| ROBUST I | 2 | 2 | 2 | 2 | 1 | 2 | 1 | 1 | 0 | 2 | 0 | 2 | **17** |
| Alhamdani 2024 | 2 | 2 | 2 | 2 | 1 | 1 | 2 | 0 | 0 | 2 | 0 | 1 | **15** |
| Noor 2024 | 2 | 2 | 2 | 2 | 2 | 2 | 2 | 0 | 0 | 2 | 0 | 2 | **18** |
| Ballesteros Ruiz 2025 | 1 | 0 | 0 | 2 | 0 | 1 | 2 | 0 | 0 | 2 | 0 | 1 | **9** |
| Van Dyke 2025 | 2 | 2 | 0 | 1 | 1 | 1 | 1 | 0 | 0 | 2 | 0 | 1 | **11** |
| Mahenthiran 2024 | 1 | 1 | 0 | 1 | 1 | 1 | 2 | 0 | 0 | 2 | 0 | 1 | **10** |

Items are scored as 0 (not reported), 1 (reported but inadequate), or 2 (reported and adequate). Score range: Minum= 0; Maximux= 24
