# Supplementary material for: Optilume, a minimally invasive solution for BPH and urethral stricture: what we know, what we need? an EAU endourology scoping review
Source: BMC Urol. 2025 Aug 9;25:196. doi: 10.1186/s12894-025-01896-3 (PMC12335074; doi:10.1186/s12894-025-01896-3)

**Supplementary Figure 1.** Risk of bias in randomized controlled trials (ROB-2).

1. Risk of bias graph: review authors' judgements about each risk of bias item presented as percentages across all included studies.


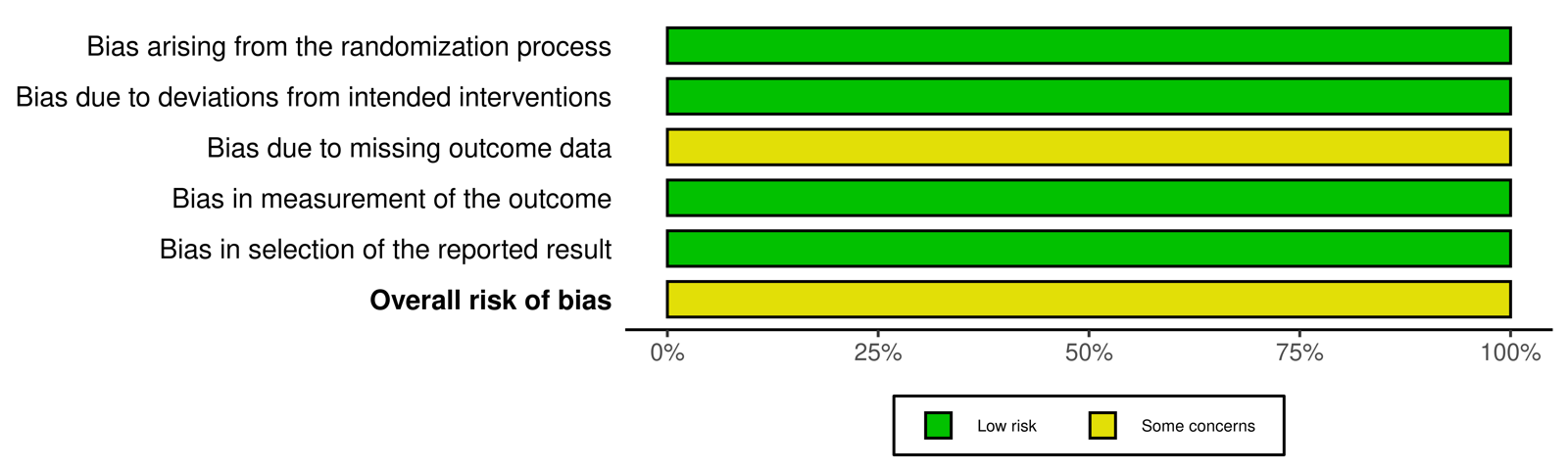


1. Risk of bias summary: review authors' judgements about each risk of bias item for each included study.


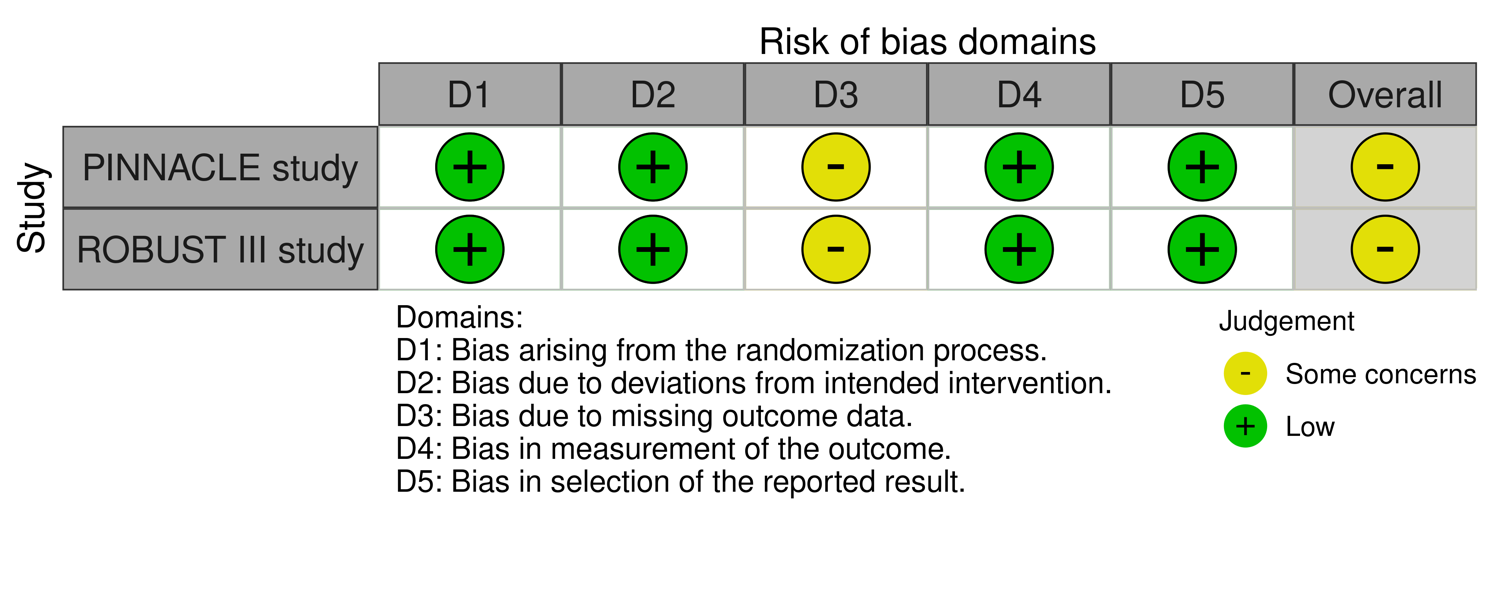

Supplement: Supplementary file 2 — Supplementary Material 2. [file 12894_2025_1896_MOESM2_ESM.docx]
